# Artificial Intelligence in Clinical Decision-Making: A Comprehensive Review of Diagnostic, Prognostic, and Therapeutic Applications, Validation Gaps, Bias, and Deployment Challenges

**DOI:** 10.7759/cureus.109905

**Published:** 2026-05-29

**Authors:** Saurabh S Patil, Gaurav Kumar Jha, Naveed Mohsin, Pankaj Patel, Lalramdinmawia Ralte, Ashish Kumar Shukla

**Affiliations:** 1 Department of Radiodiagnosis, Government Medical College, Alibagh, IND; 2 Hospital Administration, Sanjay Gandhi Postgraduate Institute of Medical Sciences, Lucknow, IND; 3 Internal Medicine, Sher-e-Kashmir Institute of Medical Sciences, Srinagar, IND; 4 Information Technology, Sardar Patel University, Vallabh Vidyanagar, IND; 5 Medical Radiology, Imaging and Therapeutic Technology, Parul Institute of Allied and Healthcare Sciences, Parul University, Vadodara, IND; 6 Radiodiagnosis, Santosh Medical College, Santosh Deemed to Be University, Ghaziabad, IND

**Keywords:** artificial intelligence, clinical decision-making, clinical decision support, deployment challenges, machine learning

## Abstract

Artificial intelligence (AI) has rapidly expanded across clinical decision-making over the past decade, with applications now reported in diagnostic imaging, risk prediction, early warning systems, treatment planning, triage, workflow optimisation, and operational decision support. This narrative comprehensive review, rather than a systematic literature review or meta-analysis, examines how AI supports clinical decision-making and what validation, implementation, bias, and deployment challenges limit its real-world use.

Literature was identified through structured searches of PubMed, Scopus, Web of Science, IEEE Xplore, and ScienceDirect for studies published between January 2015 and December 2024 using predefined AI and clinical decision-support terms. Eligible studies reported clinically relevant AI applications with clearly described datasets, modelling approaches, validation strategies, and evaluation procedures. Editorials, commentaries, letters, short abstracts, duplicates, and studies lacking sufficient methodological detail or patient-level clinical relevance were excluded. This review was not registered in International Prospective Register of Systematic Reviews (PROSPERO), did not follow a formal systematic review protocol, and did not apply a formal risk-of-bias tool such as Prediction Model Risk of Bias Assessment Tool (PROBAST) or Quality Assessment of Diagnostic Accuracy Studies-AI (QUADAS-AI); instead, methodological concerns were appraised narratively. The final narrative synthesis included 49 publications, and findings were organised by clinical domain, data source, AI approach, validation method, performance measures, implementation context, and reported limitations. Because of heterogeneity across clinical settings, datasets, model architectures, outcome definitions, comparator groups, and evaluation metrics, pooled quantitative synthesis or meta-analysis was not performed. Reported performance measures included area under the receiver operating characteristic curve (AUROC), sensitivity, specificity, accuracy, calibration, and predictive values, but these metrics were not pooled because the included studies used different populations, tasks, thresholds, and validation designs. AI showed potential benefits in imaging-based diagnosis, risk prediction, early warning systems, workflow support, and personalised treatment planning, although comparative evidence against non-AI clinical decision support or traditional statistical models was inconsistent and incompletely reported. Diagnostic AI systems were most consistently supported in imaging-based tasks such as mammography, diabetic retinopathy screening, lung nodule detection, and digital pathology, while early warning models showed promise for sepsis, acute deterioration, and critical care risk prediction. Clinical translation remains limited by retrospective and single-centre study designs, inconsistent external validation, limited prospective testing, variable calibration reporting, weak usability assessment, bias, poor transparency, and dataset shift. These limitations restrict certainty about the proportion of deployed AI systems that produce measurable clinical benefit.

AI may enhance clinical decision-making, but sustained clinical benefit requires rigorous prospective evaluation, multi-site external validation, fairness assessment, clinician-centred integration, accountable governance, and continuous post-deployment monitoring.

## Introduction and background

Artificial intelligence (AI) is increasingly used in clinical medicine to analyse healthcare data and support diagnostic, prognostic, therapeutic, and operational decision-making [1,2]. AI refers to computer-based systems that perform tasks normally requiring human intelligence, while machine learning (ML) refers to algorithms that learn patterns from data, and deep learning uses multi-layer neural networks to analyse complex data such as images, electronic health records (EHRs), and clinical text [3-5]. The increasing availability of digital health data, advances in computational capacity, and progress in algorithm development have accelerated its integration into clinical practice [3]. More recent developments, including transformer-based models, large language models, and clinical foundation models, have expanded AI applications in clinical documentation, triage, image interpretation, differential diagnosis, and decision support. However, their clinical reliability requires careful validation.

The widespread adoption of EHR systems has enabled the development of predictive models using longitudinal patient data [6,7]. However, EHR-based models may be affected by missing data, coding variation, changes in clinical practice, and limited generalisability across healthcare settings. Large clinical datasets further support model development, particularly in critical care settings [8]. In diagnostic applications, deep learning models achieve high performance in specific domains. Dermatology studies demonstrate specialist-level classification of skin cancer [9], while ophthalmology systems show strong results in diabetic retinopathy detection and screening [10,11]. In radiology, AI models are effective in breast cancer screening and lung nodule detection [12,13]. Although these findings show strong technical performance, evidence of consistent improvement in patient outcomes, workflow efficiency, and real-world clinical utility remains limited.

AI is also applied to prediction and risk stratification. Models that use EHR, laboratory, and physiological data predict outcomes such as acute kidney injury [14] and sepsis or critical-care deterioration [15,16]. Reinforcement learning methods are explored for treatment optimisation in complex conditions, including sepsis management in intensive care [17]. These applications extend AI use beyond diagnosis to early warning and clinical decision support [18]. Nevertheless, many studies remain retrospective, single-centre, and insufficiently tested in prospective or multi-institutional settings, limiting confidence in their broader clinical applicability.

Several limitations, however, constrain clinical adoption. Model performance varies across populations and healthcare settings, reflecting challenges related to generalisability and dataset shift [19,20]. Bias and unequal performance across demographic groups remain significant concerns [21,22]. Clinical usefulness depends on calibration, interpretability, and integration into existing workflows [23,24]. Reporting and evaluation frameworks aim to improve transparency and assess methodological quality in prediction models [25,26]. Ethical, legal, and regulatory issues, including accountability, privacy protection, fairness, explainability, and post-deployment monitoring, are also essential for safe implementation.

Adoption in clinical practice is influenced by clinician interaction with AI systems, including trust and workflow compatibility [23,27]. Much of the current evidence is based on retrospective analyses with limited prospective validation [19,28]. Therefore, an important gap remains between promising algorithmic performance and reliable, equitable, and clinically useful deployment in real-world healthcare systems. This review examines AI applications in clinical decision-making, with emphasis on validation, implementation, bias, transparency, governance, and clinical utility. Continued emphasis on rigorous validation, fairness, and post-deployment monitoring is required to support safe and effective use in healthcare systems [29].

Objective of the review

This review synthesises evidence of AI in clinical decision-making across diagnostic, prognostic, therapeutic, and operational domains. The review focuses on three key issues: which clinical tasks are supported by AI, how robustly these models have been externally or prospectively validated, and whether reported benefits demonstrate measurable patient or workflow outcomes rather than only surrogate performance metrics. The review also identifies barriers related to bias, transparency, regulation, workflow integration, and real-world deployment to guide safe, equitable, and clinically meaningful implementation.

Methodology

This review was conducted as a structured narrative review rather than a systematic literature review or meta-analysis of peer-reviewed studies on AI in clinical decision-making. No protocol was registered, Preferred Reporting Items for Systematic Reviews and Meta-Analyses (PRISMA) methodology was not followed, and formal search-yield or exclusion counts were not generated. Searches were performed in PubMed, Scopus, Web of Science, IEEE Xplore, and ScienceDirect for English-language studies published from January 2015 to December 2024. Search terms included “artificial intelligence,” “machine learning,” “deep learning,” “clinical decision support,” “diagnosis,” “prognosis,” “risk prediction,” “treatment planning,” “triage,” and “healthcare,” with additional terms for recent AI developments, including “transformer models,” “large language models,” “clinical foundation models,” “generative AI,” “clinical summarisation,” “differential diagnosis,” “patient-facing triage,” and “multimodal artificial intelligence.” Boolean combinations were used, and grey literature, trial registries, conference proceedings, preprint servers, and non-peer-reviewed sources were not searched. Eligible studies were peer-reviewed healthcare studies involving human healthcare data or patient-level clinical relevance and reporting AI applications for diagnosis, prediction, early warning, treatment recommendation, workflow optimisation, documentation, triage, or clinical decision support, with clear dataset and evaluation details. Editorials, commentaries, letters, short abstracts, duplicates, non-human studies, preprints, technical papers without clinical evaluation, and studies lacking methodological detail or clinical applicability were excluded. Titles and abstracts were screened for relevance, full texts of potentially eligible studies were reviewed, and 49 peer-reviewed publications were included in the final narrative synthesis. Extracted data included clinical domain, data type, modelling approach, validation strategy, performance measures, implementation context, and reported limitations. Findings were synthesised narratively across diagnostic, prognostic, therapeutic, workflow, validation, implementation, bias, transparency, and governance themes. No pooled quantitative synthesis was performed because studies differed in settings, datasets, models, outcomes, comparators, validation methods, and metrics. A formal risk-of-bias tool was not applied; methodological limitations were narratively assessed through external validation, calibration, prospective testing, dataset shift, fairness, transparency, and implementation context.

## Review

Taxonomy of AI applications in clinical decision-making

A structured taxonomy helps organise the expanding field of AI in clinical decision-making and clarifies how different systems support patient care across clinical contexts [30,31]. The taxonomy used in this review was derived from prior literature and adapted to organise the included studies according to both decision type and clinical function [29-31]. AI applications can be classified based on the type of decision supported: diagnostic, prognostic, therapeutic, and operational or workflow-related decisions [29]. Diagnostic decision support focuses on identifying disease states from imaging, laboratory, pathology, and symptom-based data, often using deep learning to extract patterns from high-dimensional inputs [31,32]. Prognostic and risk stratification systems estimate outcomes such as mortality, deterioration, readmission, or complications, supporting early intervention and clinical prioritisation [15,33]. Therapeutic decision-making includes treatment recommendations, dose optimisation, and personalised care pathways, with some models incorporating reinforcement learning or causal inference to tailor decisions to individual patients [1,17]. These categories are not mutually exclusive, and some hybrid systems may span more than one category, such as models that support both diagnosis and prognosis.

AI applications can also be categorised functionally into screening, detection, prediction, triage, monitoring, and resource allocation tasks, each with distinct performance requirements and risk profiles [8]. Screening systems prioritise sensitivity and scalability, whereas treatment planning systems require greater interpretability, uncertainty quantification, and safety considerations. This classification aligns algorithm design with validation and implementation requirements and informs regulatory and ethical evaluation [5]. Where comparative studies are available, AI and ML models do not uniformly outperform traditional statistical approaches, and their relative advantage varies according to clinical task, dataset quality, validation strategy, and implementation context [34]. It also provides a framework for comparing studies and identifying gaps relevant to clinical translation. The taxonomy used in this review was adapted from prior literature and is presented textually to organise the included studies by decision type and clinical function [29-31].

AI in diagnosis

AI has demonstrated promising but variable potential in diagnostic decision-making, particularly in imaging-based applications that rely on deep learning to process high-dimensional visual data [12,6]. In dermatology, ophthalmology, and radiology, convolutional neural networks have reported performance comparable to specialists in selected controlled tasks, such as skin cancer classification, diabetic retinopathy detection, breast cancer screening, and lung nodule identification [9,12]. AI has also been evaluated in additional diagnostic domains, including neuroimaging for stroke detection, electrocardiogram interpretation in cardiology, and emergency imaging tasks such as fracture detection, though evidence across these areas varies in validation quality and clinical maturity. These systems use large annotated datasets to learn hierarchical features directly from images, improving reproducibility and reducing reliance on manual feature engineering [27,34]. In clinical workflows, AI supports triage prioritisation, second-opinion review, and reduction of diagnostic variability in high-volume settings [15,27]. However, reported diagnostic performance should be interpreted cautiously because many studies differ in clinical task, dataset source, validation design, comparator group, threshold selection, and outcome definition.

Beyond imaging, AI is applied to laboratory data and digital pathology for disease detection and characterisation [2,35]. ML models identify abnormal patterns in structured laboratory data associated with infection, metabolic disorders, or hematologic conditions. Digital pathology systems analyse whole-slide images to detect tumours and grade malignancies with reported high sensitivity in selected study settings [33,16]. Methods such as weakly supervised learning and transfer learning improve scalability by reducing the need for extensive manual annotation [36]. Despite these advances, diagnostic AI studies often report summary performance metrics without direct head-to-head comparison across models or consistent reporting of confidence intervals, calibration, sensitivity, and specificity. Potential sources of bias include overfitting, spectrum bias, label noise, dataset imbalance, and limited external validation, which may affect reproducibility and clinical transferability.

Multimodal diagnostic models integrate imaging, laboratory data, EHRs, and genomic information into unified predictive frameworks. These approaches combine heterogeneous data sources to improve diagnostic accuracy and contextual understanding in complex clinical scenarios [9]. However, many multimodal diagnostic studies remain proof-of-concept or retrospective, and large-scale external validation in real-world clinical populations is still limited. Some studies also rely on curated, high-prevalence datasets or simulated clinical scenarios that may not reflect routine diagnostic distributions. Challenges related to data harmonisation, interoperability, and external validation across institutions remain barriers to large-scale deployment. Diagnostic AI errors also have different clinical consequences depending on the task and setting. False negatives may delay detection of serious conditions such as lung nodules, stroke, or malignancy, whereas false positives may increase unnecessary imaging, biopsy, specialist referral, anxiety, and healthcare costs. Therefore, diagnostic AI should be assessed not only by aggregate performance metrics but also by error type, clinical context, downstream decision impact, and safety monitoring. Although many studies reported favourable performance measures such as area under the receiver operating characteristic curve (AUROC), accuracy, sensitivity, specificity, and predictive values, these results were synthesised narratively rather than pooled because of substantial differences in clinical tasks, datasets, validation methods, thresholds, and outcome definitions. Therefore, claims of diagnostic AI performance in this review are interpreted cautiously and should not be read as evidence of uniform superiority across all clinical settings. Table 1 summarises key diagnostic AI applications across clinical domains.

**Table 1:** AI applications in diagnostic decision support across clinical domains AI: Artificial intelligence; EHR: Electronic health record; ML: Machine learning

Application Domain	Data Source	AI Approach	Clinical Function	Evaluation/Validation Reported	Clinical Impact or Implementation Relevance	Reference
Dermatology lesion classification	Dermoscopic images	Convolutional neural networks	Skin cancer detection	Image-based diagnostic evaluation; specialist-comparable performance reported in selected controlled datasets	May support triage and second-opinion review, but requires validation across diverse populations and clinical settings	[5]
Laboratory abnormality detection	Structured lab values	Supervised ML	Diagnostic flagging	Predictive evaluation using structured clinical data; performance depends on data quality and calibration	May support early identification of abnormal laboratory trends and clinical prioritisation	[7]
Multimodal disease diagnosis	Imaging + EHR + laboratory data	Multi-input neural networks	Integrated diagnostic support	Proof-of-concept multimodal evaluation; external validation remains limited	May improve contextual diagnostic support, but implementation depends on interoperability and data harmonisation	[9]
Diabetic retinopathy screening	Retinal fundus images	Deep learning image classifier	Automated screening	Image-classification evaluation using diagnostic performance metrics such as sensitivity, specificity, and accuracy	May support scalable screening and referral prioritisation in high-volume settings	[11]
Breast cancer detection	Mammography images	Deep neural networks	Radiology decision support	Mammography-based diagnostic validation; performance varies by dataset, threshold, and reader-comparison design	May assist radiologist decision support, but false positives and false negatives require monitoring	[12]
Lung nodule identification	Low-dose CT scans	3D deep learning models	Early cancer detection	CT-based detection evaluation; generalisability depends on validation setting and case-mix	May support early detection, but missed nodules or false-positive findings can affect downstream care	[13]
Digital pathology tumour grading	Whole-slide histopathology images	Weakly supervised deep learning	Malignancy classification	Whole-slide image evaluation; high performance reported in selected datasets, with external validation needed	May support pathology workflow and grading consistency, but deployment requires quality control and institutional validation	[33]

AI for prognosis, risk stratification, and early warning

AI has expanded clinical decision-making beyond diagnosis by enabling prediction of future health outcomes through advanced prognostic modelling [30,35]. Models based on longitudinal EHRs, physiological signals, and laboratory trends estimate individual risk profiles for mortality and complications in both inpatient and outpatient settings [7,15]. These ML and deep learning approaches capture nonlinear relationships and temporal patterns that are not easily identified using conventional statistical methods [36]. Risk stratification supports the identification of high-risk patients and enables targeted interventions and resource allocation [31]. Reported performance metrics, including AUROC, sensitivity, specificity, calibration, and predictive values, were interpreted narratively because studies varied in outcome definitions, prediction windows, validation methods, and comparator groups.

Early warning systems are increasingly used in critical care to predict sepsis, acute deterioration, and the need for intensive care escalation [15,16]. AI models analyse dynamic clinical data, including vital signs and laboratory results, to generate alerts before clinical recognition, enabling earlier intervention [16]. Deep learning approaches are also used to predict circulatory failure and haemodynamic instability from high-frequency physiological data, supporting proactive monitoring and timely response [15]. However, retrospective performance may not translate into real-world clinical benefit. External evaluations of deployed sepsis prediction tools, including the Epic Sepsis Model, have shown that model performance can decline across settings, highlighting the need for independent validation before routine use.

Predictive modelling is also applied to operational outcomes such as hospital readmission and length of stay. These models incorporate demographic characteristics, comorbidities, prior hospitalisation, and treatment history to support discharge planning and care coordination [7]. Challenges remain related to generalisability across institutions, alert fatigue, and calibration drift over time [19,20]. Outcome definition is also important, particularly for sepsis, where administrative codes, clinical criteria, and clinician judgement may identify different patient groups. False-positive alerts may increase workload and alert fatigue, whereas false-negative predictions may delay escalation of care. Temporal validation using later-period data is needed to assess whether models remain reliable after changes in protocols, coding practices, case-mix, or treatment pathways. Continuous validation and monitoring are required to maintain reliability in real-world settings. Table 2 summarises key predictive applications and modelling approaches in risk stratification.

**Table 2:** AI-based prognostic and early warning models in clinical practice Performance metrics were not pooled because studies differed in clinical settings, outcome definitions, prediction windows, validation methods, and evaluation metrics. AI: Artificial intelligence; EHR: Electronic health record; ML: Machine learning

Predictive Target	Clinical Setting	Data Type	Modeling Strategy	Evaluation/Validation Considerations	Reference
Laboratory abnormality prediction	Intensive care unit	Structured laboratory values	Deep learning models	Performance depends on data completeness, calibration, and integration with ICU workflow	[7]
Clinical outcome prediction, including acute kidney injury	Hospital/inpatient care	EHR longitudinal data	Deep learning temporal prediction model	Requires external and temporal validation because EHR data and care pathways vary across settings	[14]
Hemodynamic deterioration	Critical care	High-frequency physiological signals	Recurrent neural networks	Requires real-time data quality checks and monitoring for false alerts and calibration drift	[15]
Sepsis early detection	ICU	Vital signs + labs	Deep learning temporal models	Performance depends on sepsis definition, alert threshold, validation setting, and risk of alert fatigue	[16]
Clinical deterioration prediction, including cardiac arrest risk	Emergency department/hospital wards	EHR + triage data	Deep learning classifier	Requires prospective testing and comparison with existing clinical scoring or early warning systems	[37]
General clinical prediction modeling/ML versus traditional statistical modeling	General clinical research	Clinical datasets	Statistical vs ML models	Model usefulness depends on calibration, validation quality, interpretability, and clinical utility	[34]

AI for treatment planning and therapy optimisation

AI is increasingly applied to treatment planning by generating individualised therapeutic recommendations based on patient-specific clinical profiles and longitudinal data patterns [35,34]. ML models integrate demographic factors, comorbidities, imaging findings, laboratory results, and prior treatment responses to support personalised care strategies beyond standard protocol-based approaches [34]. However, evidence that AI-based treatment planning consistently improves outcomes beyond standard-of-care protocols remains limited, and many reported benefits relate to surrogate outcomes such as predicted response, plan quality, or model discrimination rather than patient-level clinical benefit.

In oncology, AI-based decision support tools are used for tumour classification, staging, and treatment selection across surgery, chemotherapy, radiotherapy, and immunotherapy. Predictive models analyse histopathological images, genomic data, and radiomic features to estimate treatment response and survival outcomes, supporting risk-adapted planning. AI has also been explored for drug discovery and repurposing by identifying candidate targets, compounds, and treatment combinations, although these applications remain largely preclinical or early translational. AI is also used to automate radiotherapy contouring and optimise dose distribution, improving consistency in treatment delivery [33,36]. Beyond contouring, AI methods are used for radiotherapy dose prediction, dose-volume estimation, plan-quality assessment, and adaptive planning, but clinical use requires validation across tumour sites, planning systems, and institutional protocols. Recent radiotherapy reviews similarly describe AI as promising for treatment planning and dose prediction, while emphasising the need for safe workflow integration and clinical validation.

**Figure 1:**
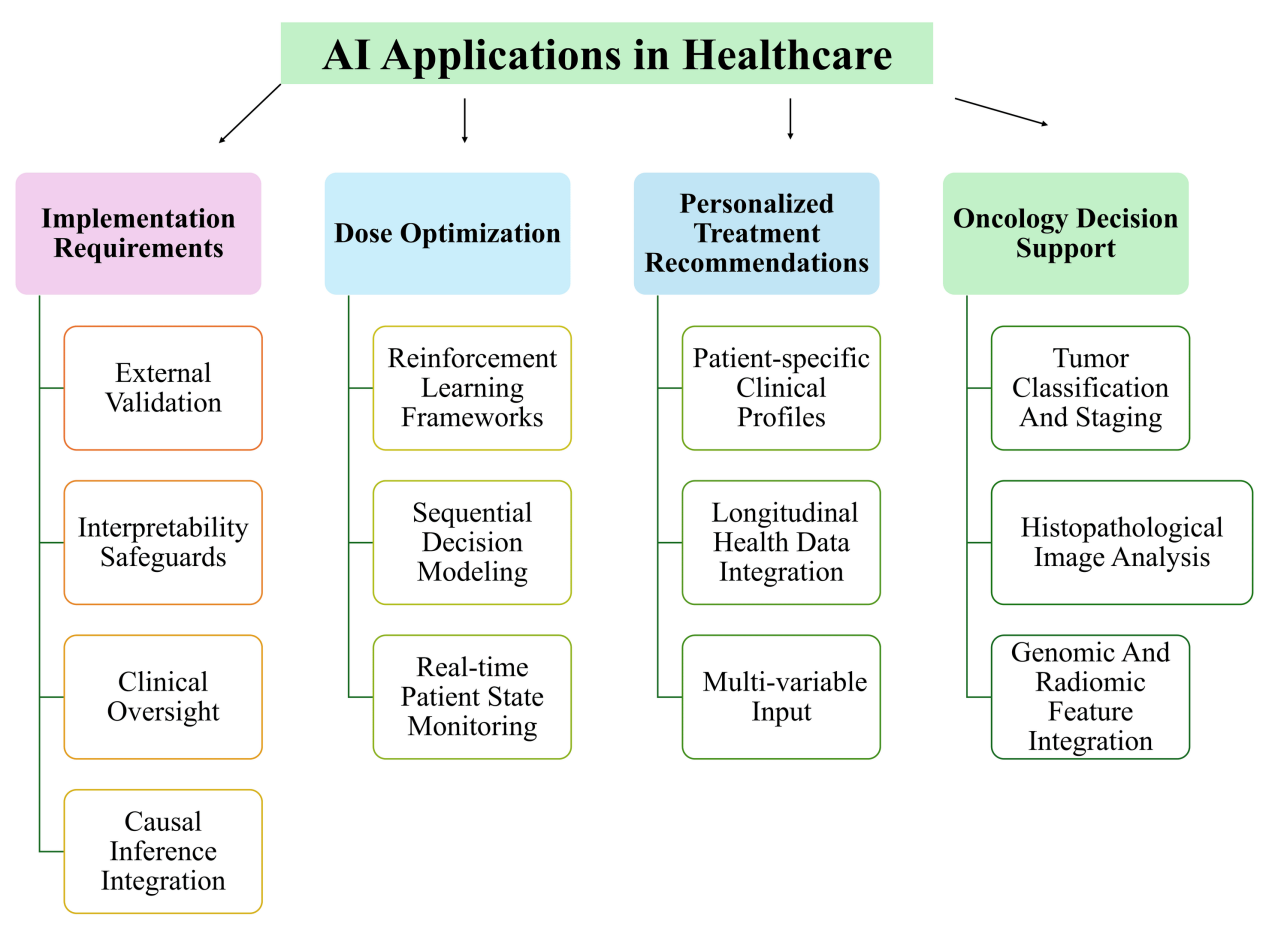
AI for treatment planning and optimisation Created by the authors using Microsoft PowerPoint AI: Artificial intelligence

AI is further applied to dose optimisation and dynamic treatment strategies, particularly in intensive care and chronic disease management. Reinforcement learning models derive data-driven treatment policies that adjust medication dosing over time based on changing patient conditions [17]. These models may identify optimal policies within historical datasets, but such policies may not be clinically actionable if they conflict with standard protocols, reflect biased treatment patterns, or lack prospective validation. Therefore, reinforcement learning recommendations should be interpreted as decision-support hypotheses rather than automatically implementable treatment instructions. Translation into clinical practice requires robust validation, interpretability, and clinical oversight to ensure safe use [19,34]. Integration of causal inference methods and real-time monitoring systems remains important for reliable implementation. Overall, treatment-planning AI should be compared with standard-of-care protocols and evaluated using patient outcomes, safety, clinician acceptance, and workflow impact before routine implementation. Figure 1 illustrates key applications of AI in treatment personalisation, oncology support, and dose optimisation.

AI clinical workflow, triage, and operational decision

AI is increasingly used to streamline clinical workflows through automation of triage, operational planning, and documentation processes within healthcare systems [27,38]. ML models for triage use presenting symptoms, vital signs, laboratory data, and clinical history to estimate acuity and risk of deterioration in emergency settings. These systems may improve triage prioritisation, reduce waiting times, and support resource allocation [9,15]. Predictive triage tools also enable early identification of patients requiring urgent care, facilitating timely intervention [39]. In emergency department operations, AI has also been applied to crowding prediction, admission forecasting, discharge planning, and waiting-time estimation, although performance depends on local patient flow, staffing patterns, seasonal demand, and data quality.

AI is also applied in operational decision-making, particularly in bed management and resource allocation. Predictive models estimate hospital admissions, length of stay, and discharge timing using historical utilisation patterns and real-time clinical data [7]. These systems support staffing decisions and improve bed utilisation, especially in resource-constrained environments. Forecasting capabilities enable planning for seasonal demand, public health emergencies, and fluctuations in elective procedures [33]. Additional operational uses include appointment scheduling, no-show prediction, patient messaging, and follow-up prioritisation. However, implementation may be limited by legacy system integration, staff acceptance, workflow disruption, and the need for local validation.

**Figure 2:**
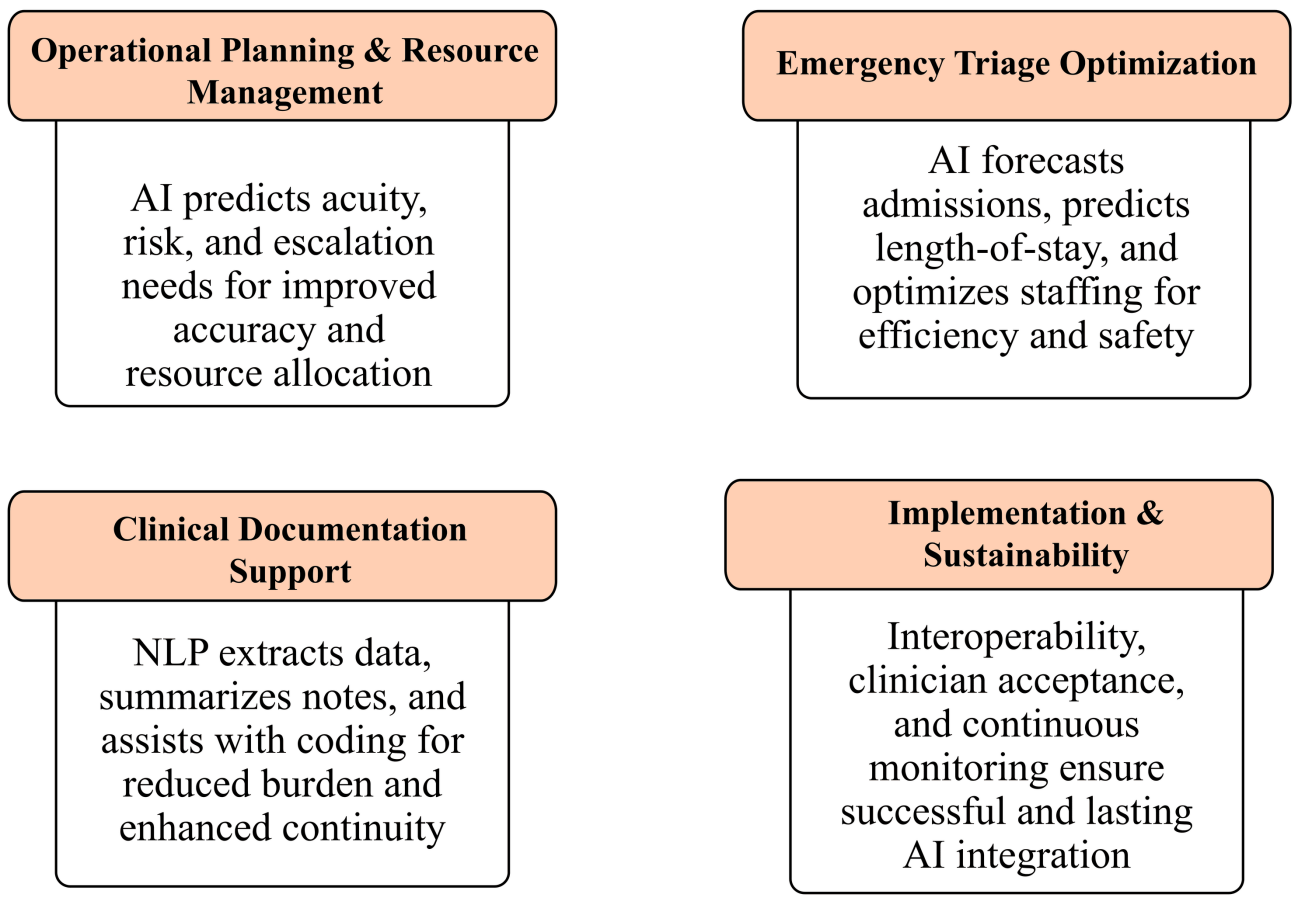
AI applications in clinical workflow, triage, and operational management Created by the authors using Microsoft PowerPoint AI: Artificial intelligence

Clinical documentation is enhanced through natural language processing techniques that extract structured information from unstructured clinical notes [15]. Applications such as automated summarisation, coding assistance, and real-time transcription reduce administrative burden and improve data quality for downstream decision support systems. NLP performance may vary across institutions because clinical notes differ in terminology, templates, abbreviations, and documentation practices. Operational AI may receive less regulatory scrutiny than diagnostic or therapeutic systems, but it can still affect patient safety through triage, access, bed allocation, discharge timing, and communication. Integration with EHR platforms supports continuity of care and accessibility of clinical information. Implementation depends on interoperability, user acceptance, and safeguards to prevent workflow disruption and unintended consequences [40]. Continuous monitoring and refinement are required to maintain reliable performance in clinical settings. Figure 2 illustrates key applications of AI in triage, operations, documentation, and implementation sustainability.

Data pipes and clinical AI modelling

Clinical AI systems rely on diverse data sources reflecting patient biology and healthcare delivery processes, with EHRs serving as a primary foundation for model development [41]. EHR datasets include structured information such as diagnoses, medications, laboratory values, and vital signs, as well as unstructured clinical narratives that can be analysed using natural language processing techniques. However, EHR data are often incomplete, irregularly recorded, and influenced by clinical workflows; missing data may reflect disease severity, testing practices, or access to care rather than random absence. Medical imaging data from radiology and pathology enable deep learning models to learn diagnostic and prognostic patterns from pixel-level representations [6,29]. Genomic and molecular data support integration of tumour biology and genetic risk into decision-making, particularly in precision medicine applications [42]. Wearable devices and remote monitoring systems provide continuous physiological data, enabling longitudinal risk prediction outside traditional clinical settings [11]. Each data source has distinct modelling challenges, including coding variation in EHRs, annotation burden in imaging, high dimensionality in genomic data, and noise or adherence issues in wearable data.

Clinical AI pipelines typically include data preprocessing, cohort definition, labelling, feature construction, model training, and evaluation. Traditional ML approaches rely on manual feature engineering, whereas deep learning methods learn representations directly from raw data. Traditional ML may be useful for smaller structured datasets and interpretable prediction tasks, while deep learning is often better suited to large imaging, waveform, text, and multimodal datasets. Adequate sample size is important for prediction model development, especially when outcomes are rare or many predictors are used, because insufficient sample size increases overfitting risk [43].

The model development lifecycle includes internal validation, external testing, calibration assessment, and monitoring to detect performance drift over time [43]. Reliable clinical translation requires clear pipeline design, transparent reporting, and specification of intended use and evaluation methods. Data leakage is a major methodological risk in clinical AI, including patient overlap between training and testing data, use of future information during prediction, or preprocessing before data splitting; these errors can inflate apparent model performance and reduce reproducibility [44]. Governance frameworks support auditing, updating, and secure deployment, ensuring continued validity as patient populations and clinical practices evolve.

Generalizability, model validation, and clinical utility

Validation is essential in clinical AI, as performance in development datasets does not ensure safe or effective use in real-world settings. External validation across institutions, populations, and time periods is required to assess generalisability. Dataset shift, arising from variations in clinical practice, data collection, and patient demographics, can degrade performance after deployment [21,44]. External validation failures have been reported when models trained or tested in one setting are applied to new institutions or populations, including sepsis prediction tools and chest X-ray models whose performance declined across hospitals. Robust evaluation, therefore, requires multi-site and temporal testing, along with calibration assessment to ensure clinically meaningful risk estimates.

Evaluation design influences the reliability of reported performance. Retrospective studies often use curated datasets and controlled labelling that may not reflect real-world complexity. Prospective evaluation provides a more accurate assessment by capturing workflow integration, clinician response, alert fatigue, and unintended consequences [44,45]. Prospective evaluation should follow recognised reporting frameworks, including Consolidated Standards of Reporting Trials-AI (CONSORT-AI) for clinical trial reports and Standard Protocol Items: Recommendations for Interventional Trials-AI (SPIRIT-AI) for clinical trial protocols involving AI interventions [46,47]. Demonstrating improvement in decision-making without increasing workload or introducing safety risks is necessary for clinical adoption. Minimum reporting and appraisal standards such as Transparent Reporting of a Multivariable Prediction Model for Individual Prognosis or Diagnosis+AI (TRIPOD+AI) and Prediction Model Risk of Bias Assessment Tool (PROBAST)+AI are also important for transparent reporting and risk-of-bias assessment in AI prediction model studies [48,49].

Clinical utility extends beyond discrimination metrics such as AUROC to include decision impact, benefit-harm balance, and patient outcomes. Decision-curve analysis estimates net benefit across threshold probabilities and determines whether a model improves decision-making compared to standard approaches [35]. Many published clinical AI studies still lack external validation, and prior reviews have reported low rates of independent external testing; therefore, reported model performance should be interpreted cautiously when validation is limited or absent. Additional metrics, including sensitivity, specificity, predictive values, calibration, fairness across subgroups, and workflow implications, are required to assess feasibility for large-scale implementation. Table 3 summarises key evaluation components for generalisability and real-world impact.

**Table 3:** Validation and clinical utility frameworks for AI systems AI: Artificial intelligence; ML: Machine learning; PROBAST: Prediction Model Risk of Bias Assessment Tool

Evaluation Component	Description	Purpose	Key Consideration	Reference
Calibration assessment	Agreement between predicted and observed risk	Clinical reliability	Probability accuracy	[20]
Human-centered clinical evaluation	Real-world assessment of AI systems in clinical workflows and user interaction	Evaluate usability, workflow impact, and patient experience	Socio-environmental factors and clinician interaction	[23]
Risk of bias and applicability assessment	Structured evaluation of prediction model bias and applicability (PROBAST domains: participants, predictors, outcome, analysis)	Detect bias and assess validity	Dataset representativeness and methodological limitations	[25]
Model development and validation pitfalls assessment	Identification of common methodological weaknesses in ML model development and validation	Improve reliability and reproducibility of prediction models	Bias in validation design, lack of calibration, and methodological shortcomings	[28]
Translation and implementation framework	Guidelines for end-to-end development and deployment of ML systems in healthcare	Enable safe and effective clinical translation	Stakeholder involvement and systematic lifecycle process	[29]
Decision-curve analysis	Net benefit estimation across decision thresholds	Clinical utility evaluation	Benefit–harm trade-off	[35]

Explainability, transparency, and trust in clinical use

Explainability is essential for AI systems used in clinical settings, where decisions directly affect patient safety and accountability. Interpretable models, such as generalised additive models and rule-based systems, provide transparent representations that allow clinicians to understand how variables contribute to predictions [40]. In contrast, complex deep learning models often rely on post-hoc explanation techniques, including feature attribution methods and saliency mapping, to approximate model reasoning. These explanations should be interpreted cautiously because they may not fully reflect how the model reached its prediction. Saliency maps should be viewed as supportive, hypothesis-generating tools rather than definitive explanations. These methods improve interpretability but may not fully represent the internal decision process, leading to discrepancies between explanations and model behaviour [37,24]. Counterfactual explanations may also help clinicians understand how changes in patient features could alter predicted risk.

Clinician trust depends on predictive accuracy, consistency, clinical plausibility, and alignment with established medical knowledge. Trust calibration refers to appropriate confidence in AI outputs, avoiding both overreliance and unwarranted scepticism. Transparent reporting of model limitations, intended use, and performance characteristics supports informed decision-making and integration into clinical workflows [24]. Providing uncertainty estimates alongside predictions allows clinicians to assess outputs probabilistically. In practice, explainability outputs should support clinical judgement, not replace it, and should be interpreted alongside the patient’s presentation and the model’s intended use.

**Figure 3:**
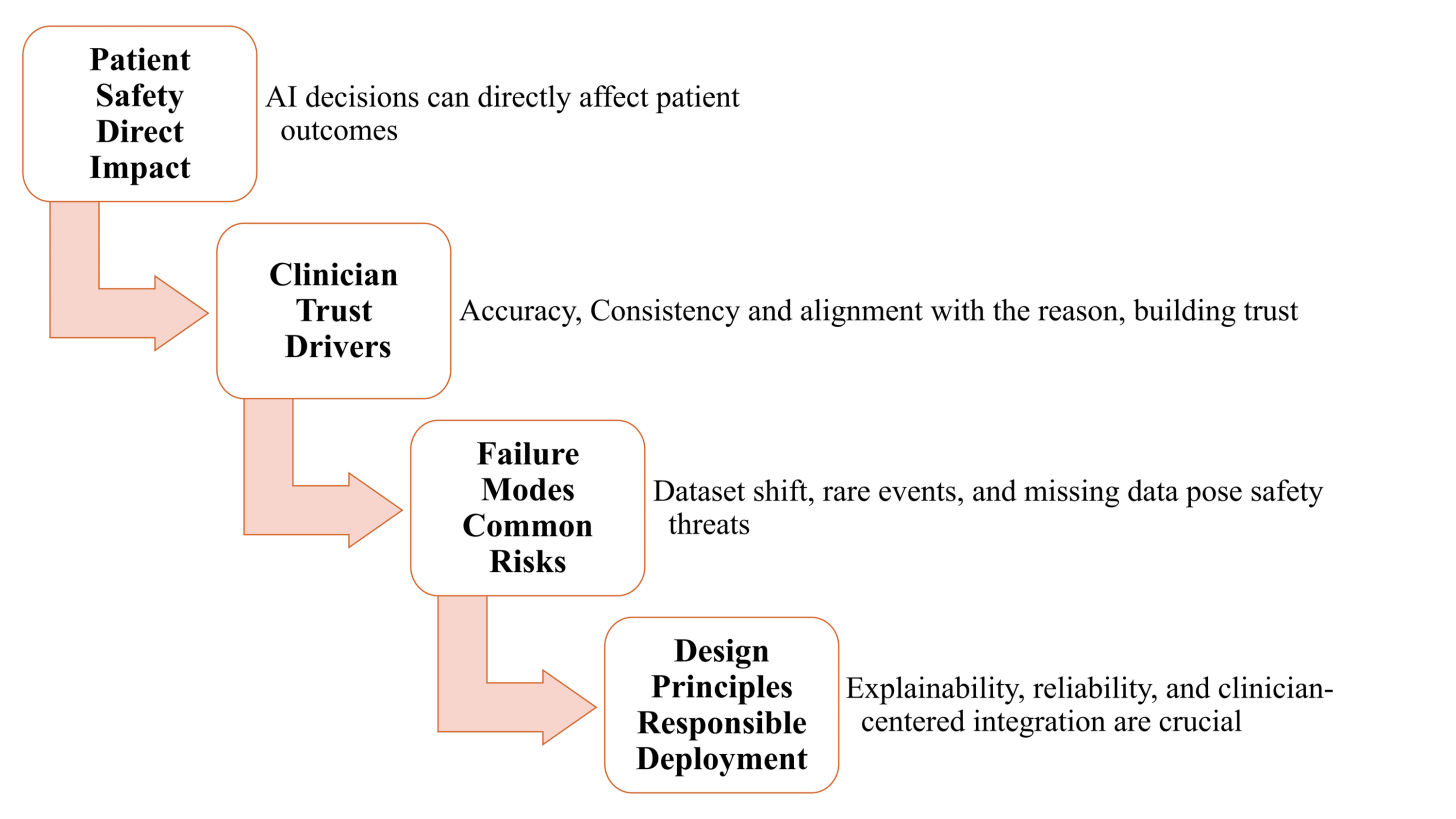
Explainability, transparency, and trust in clinical AI Created by the authors using Microsoft PowerPoint AI: Artificial intelligence

Safe deployment requires identification of potential failure modes, including performance degradation due to dataset shift, rare clinical scenarios, or missing data [21]. Methods for uncertainty quantification, such as Bayesian approaches and ensemble models, help identify low-confidence predictions and prompt human oversight. Continuous monitoring and feedback mechanisms enable detection of performance drift and support model updates [41]. Regulatory and governance expectations increasingly emphasise transparency, human oversight, performance monitoring, and clear documentation of model limitations. Emphasis on explainability, transparency, and reliability supports accountability and clinical adoption. Figure 3 illustrates key factors influencing trust and safe implementation, including patient safety, clinician confidence, and awareness of system limitations.

Ethical, legal, regulatory, and safety concerns

Ethical implementation of AI in clinical decision-making requires attention to bias, fairness, and health equity, as predictive models are trained on historical data that may reflect structural inequalities [22]. These biases can lead to disparities in performance across demographic groups, affecting access to care, diagnostic accuracy, and treatment allocation. Responsible development includes fairness-aware modelling approaches, such as bias mitigation and subgroup performance evaluation. Transparent reporting of dataset composition and evaluation across diverse populations supports accountability and appropriate use in different healthcare settings [29].

Privacy and governance are critical, as AI systems rely on large volumes of sensitive patient data from EHRs, imaging systems, and wearable devices. Ensuring data confidentiality requires secure data handling, de-identification, and compliance with data protection regulations. Governance frameworks should define data ownership, access controls, and monitoring mechanisms to support ethical data use throughout the model lifecycle.

Regulatory frameworks for AI-based medical systems address adaptive algorithms used in clinical decision support. Agencies such as the Food and Drug Administration (FDA) and European Medicines Agency (EMA) guide safety, performance, and lifecycle management [8]. Clinical liability remains complex, particularly when decision-making is shared between clinicians and AI systems. Responsibility for harm may involve multiple parties, including clinicians who use the recommendation, healthcare institutions that deploy the system, and developers or manufacturers responsible for model design, validation, and monitoring. Clear local policies are therefore needed to define oversight, escalation pathways, documentation requirements, and accountability before clinical deployment. Post-deployment monitoring is required to assess real-world performance, detect calibration drift, and identify unintended harms [33]. Ongoing model review and updating are necessary to maintain clinical validity as patient populations and healthcare practices evolve.

Limitations of the current evidence base

The existing literature on AI in clinical decision-making has several important limitations in scope, methodology, and generalisability. Most studies use retrospective, single-centre designs, which limit generalisability to diverse healthcare settings. There is a strong emphasis on discrimination metrics, while calibration, workflow integration, patient-centred outcomes, and cost-effectiveness receive less attention. Inconsistent reporting of preprocessing methods and labelling criteria further limits reproducibility. Additional limitations include publication bias, limited comparator studies against standard care or traditional risk scores, poor representation of low-resource settings, limited patient and public involvement, insufficient long-term safety monitoring, and the potential for clinician overreliance or deskilling. Deployment barriers, particularly interoperability with existing EHR systems and performance degradation due to dataset shift, remain inadequately addressed in the literature.

Future directions

Improving generalisability requires multi-institutional external validation across at least two distinct health systems, preferably with different patient demographics and practice patterns. Future research should prioritise prospective and, where appropriate, randomised studies that assess patient outcomes, workflow impact, safety, quality of care, and patient-reported outcomes rather than relying only on retrospective AUROC or accuracy estimates. Validation practices should adopt established reporting standards such as TRIPOD-AI and Developmental and Exploratory Clinical Investigations of Decision-Support Systems Driven by Artificial Intelligence (DECIDE-AI), with calibration assessment and decision-curve analysis where appropriate. Implementation science studies are needed to examine workflow integration, clinician adoption, alert fatigue, institutional readiness, and unintended consequences. Model development should include fairness-aware learning approaches, uncertainty quantification, and interpretable or post-hoc explainability methods, with empirical evaluation of clinician trust calibration. Health equity assessments should include subgroup analyses across demographic, socioeconomic, and clinical groups. Cost-effectiveness and comparative effectiveness studies are also needed to determine whether AI provides added value over standard care, traditional risk scores, or non-AI clinical decision support. Clinician training should support safe AI use, appropriate interpretation of outputs, and maintenance of clinical competency. Safety requires continuous post-deployment auditing with prespecified performance monitoring metrics, automated drift detection, and governance-defined criteria for model retraining or retirement. Advances in multimodal data integration, EHR data linkage, and causal inference methods hold promise for improving treatment personalisation and dynamic optimisation.

## Conclusions

This review concludes that AI may support clinical decision-making across diagnostic, prognostic, therapeutic, medication management, and healthcare operations domains. AI models have shown promising performance in imaging-based diagnosis, early warning systems, risk stratification, targeted therapy optimisation, and clinical workflow assistance. However, clinical translation remains inconsistent because many studies are retrospective, single-centre, and limited by insufficient external validation, dataset shift, limited interpretability, and weak evidence of patient-centred benefit. Ethical and governance concerns, including bias, fairness, privacy, accountability, and safety monitoring, remain central to responsible implementation. Regulatory and reporting frameworks such as TRIPOD+AI, PROBAST+AI, CONSORT-AI, SPIRIT-AI, and DECIDE-AI can strengthen transparency, evaluation quality, and clinical accountability. Future progress should prioritise clinician-centred workflow integration, multisite prospective evaluation, transparent reporting, continuous post-deployment monitoring, and governance frameworks that ensure safety as patient populations and clinical practices evolve. Multimodal data fusion and uncertainty-aware models may further improve clinical relevance by communicating confidence and supporting safer decision-making. AI offers substantial potential to improve clinical decision-making, but this potential will be realised only through rigorous evaluation, equity-focused development, and sustained accountability.

## References

[1] Topol EJ (2019). High-performance medicine: the convergence of human and artificial intelligence. Nat Med.

[2] Yu KH, Beam AL, Kohane IS (2018). Artificial intelligence in healthcare. Nat Biomed Eng.

[3] He J, Baxter SL, Xu J, Xu J, Zhou X, Zhang K (2019). The practical implementation of artificial intelligence technologies in medicine. Nat Med.

[4] Chen JH, Asch SM (2017). Machine learning and prediction in medicine - beyond the peak of inflated expectations. N Engl J Med.

[5] Esteva A, Kuprel B, Novoa RA, Ko J, Swetter SM, Blau HM, Thrun S (2017). Dermatologist-level classification of skin cancer with deep neural networks. Nature.

[6] Erickson BJ, Korfiatis P, Akkus Z, Kline TL (2017). Machine learning for medical imaging. Radiographics.

[7] Ayad A, Hallawa A, Peine A (2022). Predicting abnormalities in laboratory values of patients in the intensive care unit using different deep learning models: comparative study. JMIR Med Inform.

[8] Johnson AE, Pollard TJ, Shen L (2016). MIMIC-III, a freely accessible critical care database. Sci Data.

[9] Soenksen LR, Ma Y, Zeng C (2022). Integrated multimodal artificial intelligence framework for healthcare applications. NPJ Digit Med.

[10] De Fauw J, Ledsam JR, Romera-Paredes B (2018). Clinically applicable deep learning for diagnosis and referral in retinal disease. Nat Med.

[11] Ting DS, Cheung CY, Lim G (2017). Development and validation of a deep learning system for diabetic retinopathy and related eye diseases using retinal images from multiethnic populations with diabetes. JAMA.

[12] McKinney SM, Sieniek M, Godbole V (2020). International evaluation of an AI system for breast cancer screening. Nature.

[13] Ardila D, Kiraly AP, Bharadwaj S (2019). End-to-end lung cancer screening with three-dimensional deep learning on low-dose chest computed tomography. Nat Med.

[14] Tomašev N, Glorot X, Rae JW (2019). A clinically applicable approach to continuous prediction of future acute kidney injury. Nature.

[15] Hyland SL, Faltys M, Hüser M (2020). Early prediction of circulatory failure in the intensive care unit using machine learning. Nat Med.

[16] Nemati S, Holder A, Razmi F, Stanley MD, Clifford GD, Buchman TG (2018). An interpretable machine learning model for accurate prediction of sepsis in the ICU. Crit Care Med.

[17] Komorowski M, Celi LA, Badawi O, Gordon AC, Faisal AA (2018). The artificial intelligence clinician learns optimal treatment strategies for sepsis in intensive care. Nat Med.

[18] Sutton RT, Pincock D, Baumgart DC, Sadowski DC, Fedorak RN, Kroeker KI (2020). An overview of clinical decision support systems: benefits, risks, and strategies for success. NPJ Digit Med.

[19] Kelly CJ, Karthikesalingam A, Suleyman M, Corrado G, King D (2019). Key challenges for delivering clinical impact with artificial intelligence. BMC Med.

[20] Van Calster B, McLernon DJ, van Smeden M, Wynants L, Steyerberg EW (2019). Calibration: the Achilles heel of predictive analytics. BMC Med.

[21] Vokinger KN, Feuerriegel S, Kesselheim AS (2021). Mitigating bias in machine learning for medicine. Commun Med (Lond).

[22] Perez-Downes JC, Tseng AS, McConn KA (2024). Mitigating bias in clinical machine learning models. Curr Treat Options Cardio Med.

[23] Beede E, Baylor E, Hersch F, Iurchenko A, Wilcox L, Ruamviboonsuk P, Vardoulakis LM (2020). A human-centered evaluation of a deep learning system deployed in clinics for the detection of diabetic retinopathy. Proc CHI Conf Hum Factors Comput Syst.

[24] Markus AF, Kors JA, Rijnbeek PR (2021). The role of explainability in creating trustworthy artificial intelligence for health care: a comprehensive survey of the terminology, design choices, and evaluation strategies. J Biomed Inform.

[25] Wolff RF, Moons KG, Riley RD (2019). PROBAST: A tool to assess the risk of bias and applicability of prediction model studies. Ann Intern Med.

[26] Collins GS, Reitsma JB, Altman DG, Moons KG (2015). Transparent reporting of a multivariable prediction model for individual prognosis or diagnosis (TRIPOD): the TRIPOD statement. Ann Intern Med.

[27] Abràmoff MD, Lavin PT, Birch M, Shah N, Folk JC (2018). Pivotal trial of an autonomous AI-based diagnostic system for detection of diabetic retinopathy in primary care offices. NPJ Digit Med.

[28] Roberts M, Driggs D, Thorpe M (2021). Common pitfalls and recommendations for using machine learning in healthcare. Nat Mach Intell.

[29] Wiens J, Saria S, Sendak M (2019). Do no harm: a roadmap for responsible machine learning for health care. Nat Med.

[30] Rajpurkar P, Chen E, Banerjee O, Topol EJ (2022). AI in health and medicine. Nat Med.

[31] Obermeyer Z, Emanuel EJ (2016). Predicting the future - big data, machine learning, and clinical medicine. N Engl J Med.

[32] Esteva A, Robicquet A, Ramsundar B (2019). A guide to deep learning in healthcare. Nat Med.

[33] Campanella G, Hanna MG, Geneslaw L (2019). Clinical-grade computational pathology using weakly supervised deep learning on whole slide images. Nat Med.

[34] Christodoulou E, Ma J, Collins GS, Steyerberg EW, Verbakel JY, Van Calster B (2019). A systematic review shows no performance benefit of machine learning over logistic regression for clinical prediction models. J Clin Epidemiol.

[35] Kerr KF, Brown MD, Zhu K, Janes H (2016). Assessing the clinical impact of risk prediction models with decision curves: guidance for correct interpretation and appropriate use. J Clin Oncol.

[36] Efthimiou O, Seo M, Chalkou K, Debray T, Egger M, Salanti G (2024). Developing clinical prediction models: a step-by-step guide. BMJ.

[37] Kwon JM, Lee Y, Lee Y, Lee S, Park J (2018). An algorithm based on deep learning for predicting in-hospital cardiac arrest. J Am Heart Assoc.

[38] Dandolo D, Masiero C, Carletti M, Dalle Pezze D, Susto GA (2023). AcME - accelerated model-agnostic explanations: fast whitening of the machine-learning black box. Expert Syst Appl.

[39] Thomas DM, Kleinberg S, Brown AW (2022). Machine learning modeling practices to support the principles of AI and ethics in nutrition research. Nutr Diabetes.

[40] Mateen BA, Liley J, Denniston AK, Holmes CC, Vollmer SJ (2020). Improving the quality of machine learning in health applications and clinical research. Nat Mach Intell.

[41] Morley J, Machado CC, Burr C, Cowls J, Joshi I, Taddeo M, Floridi L (2020). The ethics of AI in health care: a mapping review. Soc Sci Med.

[42] Riley RD, Ensor J, Snell KI (2020). Calculating the sample size required for developing a clinical prediction model. BMJ.

[43] Riley RD, Snell KI, Ensor J, Burke DL, Harrell FE Jr, Moons KG, Collins GS (2019). Minimum sample size for developing a multivariable prediction model: PART II - binary and time-to-event outcomes. Stat Med.

[44] Kapoor S, Narayanan A (2023). Leakage and the reproducibility crisis in machine-learning-based science. Patterns (N Y).

[45] Vasey B, Nagendran M, Campbell B (2022). Reporting guideline for the early stage clinical evaluation of decision support systems driven by artificial intelligence: DECIDE-AI. BMJ.

[46] Liu X, Cruz Rivera S, Moher D, Calvert MJ, Denniston AK (2020). Reporting guidelines for clinical trial reports for interventions involving artificial intelligence: the CONSORT-AI extension. Lancet Digit Health.

[47] Cruz Rivera S, Liu X, Chan AW, Denniston AK, Calvert MJ (2020). Guidelines for clinical trial protocols for interventions involving artificial intelligence: the SPIRIT-AI extension. Lancet Digit Health.

[48] Collins GS, Moons KG, Dhiman P (2024). TRIPOD+AI statement: updated guidance for reporting clinical prediction models that use regression or machine learning methods. BMJ.

[49] Moons KG, Damen JA, Kaul T (2025). PROBAST+AI: an updated quality, risk of bias, and applicability assessment tool for prediction models using regression or artificial intelligence methods. BMJ.

